# Properties of Controlled Low Strength Material with Circulating Fluidized Bed Combustion Ash and Recycled Aggregates

**DOI:** 10.3390/ma11050715

**Published:** 2018-05-02

**Authors:** Wei-Ting Lin, Tsai-Lung Weng, An Cheng, Sao-Jeng Chao, Hui-Mi Hsu

**Affiliations:** 1 Department of Civil Engineering, National Ilan University, No.1, Sec. 1, Shennong Rd., I-Lan 260, Taiwan; ancheng@niu.edu.tw (A.C.); chao@niu.edu.tw (S.-J.C.); hmhsu@niu.edu.tw (H.-M.H.); 2 Physics Division, Tatung University, No.40, Sec. 3, Zhongshan N. Rd., Taipei 104, Taiwan; wengabc@ttu.edu.tw; 3 Institute of Engineering Management, College of Engineering, Tatung University, No.40, Sec. 3, Zhongshan N. Rd., Taipei 104, Taiwan

**Keywords:** CFBC ash, CLSM, slag, green materials

## Abstract

This study aims to investigate the effect of adding circulating fluidized bed combustion (CFBC) ash, desulfurization slag, air-cooled blast-furnace slag and coal bottom ash to the controlled low-strength material (CLSM). Test methods include slump flow test, ball drop test, water soluble chloride ion content measurement, compressive strength and length change measurement. The results show that (1) the use of CFBC hydration ash with desulfurization slag of slump flow is the best, and the use of CFBC hydration ash with coal bottom ash and slump flow is the worst; (2) CFBC hydration ash with desulfurization slag and chloride ion content is the highest; (3) 24 h ball drop test (diameter ≤ 76 mm), and test results are 70 mm to 76 mm; (4) CFBC hydration ash with desulfurization slag and compression strength is the highest, with the coal bottom ash being the lowest; increase of CFBC hydration ash can reduce compressive strength; and (5) the water-quenched blast furnace slag and CFBC hydration ash would expand, which results in length changes of CLSM specimens.

## 1. Introduction

Flourishing economic development, rapid increase in population, and the vigorous promotion of various public works in Taiwan in recent years have led to the heavy consumption of cement materials. Cement production generates a fair amount of pollution and produces a ton of carbon dioxide for every cubic meter of cement consumed. For the sake of environmental protection, gradually reducing cement consumption and searching for suitable replacements for cement have become burning issues in recent years.

To address the growing amount of pollution in the environment, new combustion technologies were sought, which resulted in the introduction of circulating fluidized bed combustion (CFBC) technology. CFBC has been successfully applied to reuse industry by-products as well as combustible or mixed fuels in circulating fluidized beds (CFB) [1,2]. It also was a combustion technology that quickly developed in the last two decades and offers high efficiency and low pollution (less NO_x_ and SO_2_ emissions) [3,4]. It offers high efficiency, low pollution, good coal adaptability, strong load adjustment capacity, relatively low costs, and relatively easy-to-grasp technology [5,6,7]. In another aspect, gravel excavation is limited in Taiwan, thereby creating an imbalance in supply and demand. For this reason, developing aggregate alternatives and promoting the utilization of industry by-products can effectively benefit the conservation of limited natural resources and reduce the consumption of raw materials in cement industry.

The ash products of CFB processes are stable and do not leach toxic substances. Thus, they can be used in road paving, soil improvement, and roadbed filling. Depending on where the by-product lime is collected during CFB processes and how they are processed, CFBC ashes can be divided into the following three categories [8,9]:

Fly ash (powders): This type of ash is collected using bag filters in CFBs. A yellowish brown powder is mainly comprised of anhydrous calcium sulfate as well as some calcium oxide and calcium hydroxide. Its specific weight is approximately 2.80; 93% of it can pass through a #200 mesh, and its fineness ranges from 2884 cm^2^/g to 3050 cm^2^/g. Its primary uses include raw material for soil conditioners, controlled low-strength materials (CLSMs), and plasterboard in addition to being a dehydration curing agent and an alkali activator.
Bed ash (granules): This is collected from the bottoms of boilers. It comprises yellowish brown granules mixed with some black and white impurities. Particle sizes range from 0.6 mm to 0.075 mm, and its specific gravity (g/cm^3^) and fineness are 3.05 and 1260 cm^2^/g, respectively. In size, it resembles fine sand, and, in composition, it is close to fly ash. Its primary uses include raw material for by-product lime fertilizers and plasterboard.
Hydrated ash (hydrous): Comprising dark gray caked particles, hydrated ash is the result of CFBC fly ash and bed ash mixed in water for hydration, soaked for roughly 24 h, and then sun-dried. Soaked in water, the anhydrous calcium sulfate is hydrated into gypsum. It has a bulk density between 1200 kg/m^3^ and 1700 kg/m^3^, California bearing ratio (CBR) greater than 85%, maximum dry density 1414 kg/m^3^, expansion rate 0.08%, and optimum water content 28 ± 2%. It is mainly used in aggregate grading and landfill material.

The fuels of CFBC are generally high-sulphur substances. For the sake of desulfurization, large quantities of limestone are added, which means greater sulfur and calcium oxide content. The main products are CaSO_3_ and free-CaO [1]. Anthony (2002) and Qian (2006) [10,11] indicated that using free-CaO as the alkali activator of SiO_2_ and Al_2_O_3_ produces C-S-H and C-A-H gels. Excessive amounts of SO_3_ and free-CaO in cementitious material systems can have adverse effects on strength development and volume stability. Using cement and fly ash from pulverized coal as cementing material and CaSO_4_ as an activator, Poon examined the influence of adding CaSO_4_ to cement-fly ash systems on compressive strength [12]. Test results revealed that adding CaSO_4_ had a desirable effect on early strength, especially in systems with greater fly ash content. Furthermore, the amount of ettringite produced increased with the amount of cement replaced by CFBC ash. Sheng discovered that higher f-CaO and SO_3_ contents facilitate the formation of ettringite and C-S-H gel and enhance early strength [13]. Ground CFBC ash has significant impact on cementation, with greater fineness resulting in greater compressive strength [14,15]. Desulfurized slag is general industrial waste in Taiwan and classified as a reusable waste by the Environmental Protection Administration [16]. It is a solid waste produced by the desulphurization of hot metal in blast furnaces at steelworks where iron ore is the primary raw material. At present, the only manufacturer of desulfurized slag in Taiwan is the China Steel Company, which yields roughly 250 thousand tons every year.

Controlled low-strength material (CLSM) is a cementitious material that is mainly used as a backfill for roads or pipelines. It is also defined by American Society for Testing and Materials (ASTM) D4832 as “a mixture of soil, cementitious materials, water, and sometimes admixtures, that hardens into a material with a higher strength than the soil but less than 1200 psi” and by ASTM D5971 as “a self compacting, flowable, cementitious material that is primarily used as a backfill or structural fill instead of compacted fill or unsuitable native soil” as well as “a non-flowable compacted material or as a mortar”. According to American Concrete Institute (ACI) 229R, the basic composition of CLSM is Portland cement, fly ash, chemical admixtures, water, aggregates, and non-standard materials. According to ASTM D5971, it is comprised of Portland cement, fly ash, aggregates, water, and chemical admixtures. In practical application in Taiwan, the uses and composition of CLSM are significantly different from those of general concrete, containing coarse and fine aggregates, Portland cement, and water. General concrete is subject to strict restrictions with regard to the particle size distribution of coarse and fine aggregates and organic content. No particular restrictions exist for CLSM, so it can contain recycled aggregates such as discarded brick, blast furnace slag, and foundry sand [17,18,19,20,21]. The recycling and reuse of the combined bottom ash could help to alleviate the problem of inadequate landfill space and reduce secondary pollution.

The production of natural aggregate in Taiwan is somewhat limited; therefore, to facilitate energy conservation, environmental protection, and sustainable development, this study used CFBC hydrated ash, coal bottom ash, desulfurized slag, and air-cooled blast furnace slag as fully aggregate replacements and mixed CFBC ash in CLSM and examined their influence on CLSM properties using the slump flow test, the ball drop test, water-soluble chloride ion content, length variations, and the compressive strength test. The progressive strength development observed in this study may have originated from the low reactivity of the calcium source in the CFBC by-product ashes and slags that enabled the formation of reaction products, providing the reactants and increasing the strength of the CLSM materials.

## 2. Materials and Methods 

### 2.1. Materials

This study examined the influence of fully replacing of natural aggregates using various proportions of recycled aggregates on the properties of CLSM. The primary recycled aggregate variable was CFBC hydrated ash, which we used to replace 15%, 25%, 35%, and 45% of the natural aggregates. We then paired these proportions with coal bottom ash, air-cooled blast furnace slag, and desulfurized slag as recycled aggregates. It was used Type I Portland cement from the Taiwan Cement Company and water-quenched blast furnace slag produced by the CHC Resources Corporation (Kaohsiung, Taiwan). The specific gravity (g/cm^3^) and fineness of the latter were 2.83 and 6000 cm^2^/g, respectively. The CFBC fly ash, which mainly consisted of anhydrous calcium sulfate, was produced by the Formosa Plastics Company with petroleum coke as the fuel and had a specific gravity (g/cm^3^) and fineness of approximately 2.80 and 3000 cm^2^/g. Originating from the Formosa Plastics Group (Yunlin, Taiwan), the CFBC hydrated ash was the hydrated mixture of CFBC fly ash and bottom ash and mostly contained gypsum; its specific gravity (g/cm^3^), fineness modulus (FM), and water-soluble chloride ion content were 1.88, 3.96, and 0.0143%, respectively. Table 1 displays the detailed composition as conducted according to ASTM C114.

**Figure 1:**
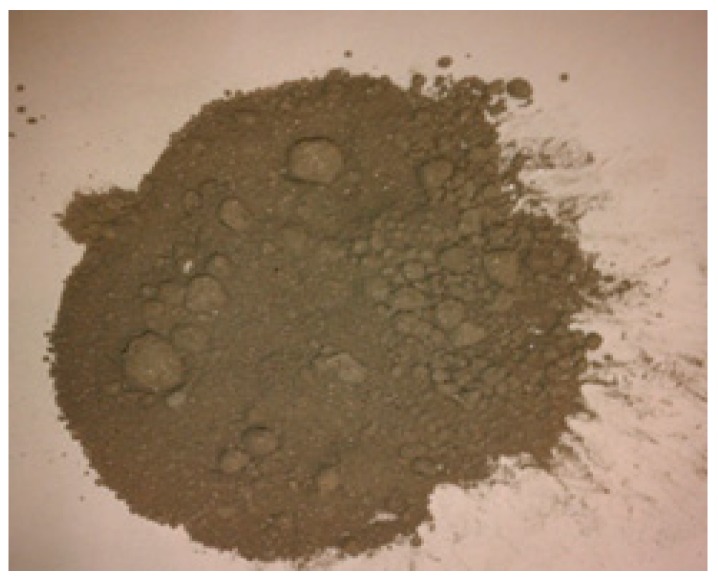
Desulfurized slag.

**Figure 2:**
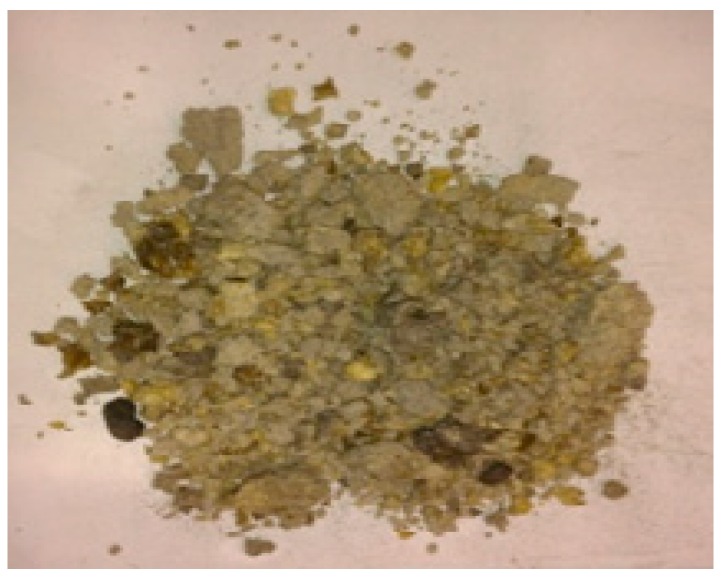
Coal bottom ash.

**Figure 3:**
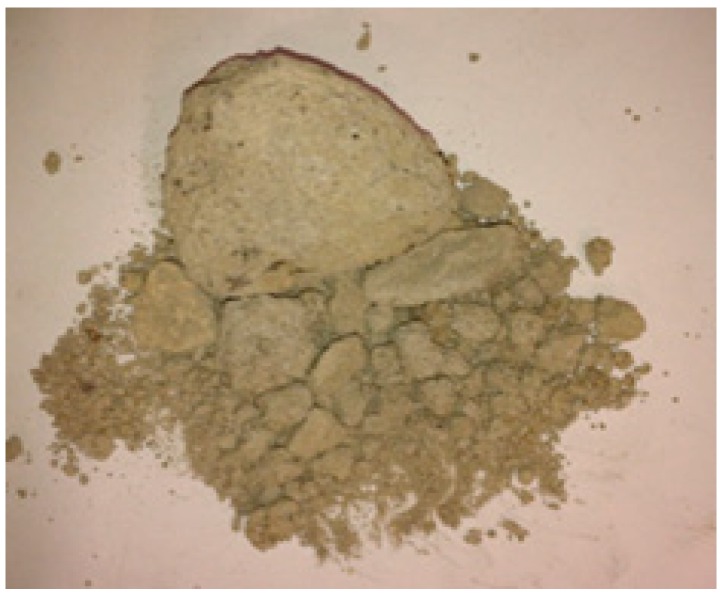
Air-cooled blast furnace slag.

Displayed in Figure 1, the desulfurized slag was produced from the desulfurization of molten iron ore. Its specific gravity (g/cm^3^), water absorption rate, FM, and water-soluble chloride ion content were 2.5, 21.3%, 2.73, and 0.17%. The primary components of coal bottom ash, shown in Figure 2, were SiO_2_, Al_2_O_3_, Fe_2_O_3_, and CaO; its specific gravity (g/cm^3^), FM, and water-soluble chloride ion content were 1.78, 3.41, and 0.0017%, respectively. The specific gravity (g/cm^3^), water absorption rate, FM, and water-soluble chloride ion content of the air-cooled blast furnace slag, exhibited in Figure 3, were 2.01, 4.17%, 6.28, and 0.0012%. The grain size of the desulfurized slag and coal bottom ash passing through sieve from #100 (150 μm) to #4 (4.75 mm) were used; the air-cooled blast furnace slag was used from #100 (150 μm) to 3/2” (37.5 mm)

### 2.2. Tested Mixtures

The fixed variables in the CLSM mixtures in this study include water–binder ratio 0.69, superplasticizer content 2%, water-quenched blast furnace slag 210 kg/m^3^, cement 60 kg/m^3^, and CFBC fly ash 30 kg/m^3^. Table 2 presents all of the tested mixtures. The specimens were numbered as follows. The digit indicates the proportion of recycled aggregates using CFBC hydrated ash, with 1, 2, 3, and 4 representing 15%, 25%, 35%, and 45%, respectively. The letter indicates the recycled aggregate composition; A signifies mixtures comprising CFBC hydrated ash, coal bottom ash, desulfurized slag, and air-cooled blast furnace slag; B represents mixtures of CFBC hydrated ash and coal bottom ash; C indicates mixtures of CFBC hydrated ash and air-cooled blast furnace slag, and D represents mixtures containing CFBC hydrated ash and desulfurized slag.

### 2.3. Test Methods and Specimens

The slump flow test was performed according to China National Standard (CNS) 14842 (Slump Flow Test Method for High Fluidity Concrete). Fresh CLSM mixtures were first poured into the slump cone with no layering or tamping. Once the slump cone was full, the top was scraped flat, and then the cone was lifted vertically. When the fresh CLSM mixture stopped flowing, the diameters of the mixture at various angles were measured using a tape measure and then averaged. The purpose of this test was to determine whether the tested CLSM mixtures conformed to the regulation stipulating that slump flow must reach 40 cm [22].

Water-soluble chloride ion content was measured in accordance with CNS 13465 (Measurement Method for Water-soluble Chloride Ion Content in Fresh Concrete) and CNS 13407 (Measurement Method for Water-soluble Chloride Ion Content in Fine Aggregates) [23]. The chloride ion content of fresh CLSM mixtures was measured using a DY-2501 chloride meter (Daeyoon Scale Industrial Co. Ltd., Seoul, Korea). The probe of the meter was first placed in a calibration solution for calibration. Then, the probe was set in a fresh CLSM mixture for several minutes. The chloride content was measured several times for each mixture, the results of which were averaged to minimize the error. However, relevant regulations in Taiwan stipulate that this test be performed in accordance with CNS 13465 and that all results must conform to the standards in CNS 3090. If there is no concern of steel corrosion, then this test can be foregone with engineer approval. The purpose of this test was to understand whether the chloride ion contents of the fresh CLSM mixtures are lower than the stipulated 0.15 kg/m^3^ for pre-stressed concrete and 0.30 kg/m^3^ for reinforced concrete, which is based on the above standards.

The ball drop test was conducted according to ASTM D6024. The ball drop impact tester was placed over the specimen and immediately released at the high point. This was repeated five times, and then the average diameter of the indentation was measured using a tape measure and the diameter of the indentations was smaller than 76 mm. This test is necessary as CLSM is primarily used as backfill for roads. Ball drop tests can be aimed at general or early strength. The former are performed between 12 h and 24 h, whereas the latter are performed between 3 h and 4 h.

Compressive strength was gauged in accordance with ASTM D4832. Fresh CLSM mixtures were first poured into φ10 cm × 20 cm molds. After solidifying, the specimens were demolded and cured in a curing tank for 28 days. Then, they were removed from the tank, dried with a rag, and capped with gypsum. Their compressive strength was tested using a universal tester, with compression time no less than 2 min. This test was based on the basic definition in ACI 116R, stating that CLSM is a material with a compressive strength no greater than 1200 psi (8.24 MPa = 84 kgf/cm^2^). If the possibility of future re-excavations is considered, then the compressive strength must be 300 psi (2.10 MPa = 21.41 kgf/cm^2^) or less. As for CLSM used as the structural backfill, adopting compressive strength of 1200 psi removes the option of re-excavation after construction is completed. The construction guidelines provided by Public Construction Commission of the Executive Yuan recommends that the compressive strength of CLSM be 90 kgf/cm^2^ (8.82 MPa) or less.

Variations in length were measured according to the drying shrinkage test method given in ASTM C227. The purpose of this test was to understand variations in CLSM specimen length at 28 days. The dimensions of the CLSM specimens were 285 mm × 75 mm × 75 mm. The CLSM mixtures were poured into the molds and demolded at 24 ± 0.5 h. In the event of inadequate strength at demolding, the demolding time was changed to 48 ± 0.5 h. Initially values were measured upon demolding, and then the specimens were cured and tested at 7 days, 14 days, and 28 days. Three specimens were tested for each mixture in each test, and then the results were averaged and compared. A standard deviation was controlled less than 10% for the tested results.

## 3. Results

### 3.1. Slump Flow

CLSM has high flowability and workability and the results are presented in Table 3. As can be seen, all of the mixtures displayed slump flow values greater than 40 cm, which conformed to the performance requirements for general flowability grade for CLSM. The slump flow values of Specimens 1D, 2D, 3D, and 4D were the highest. This is because, as explained in the previous study [24], the grading and shapes of aggregates exert impact on the physical properties of CLSM. These physical properties include flowability and compressive strength. Evenly graded aggregates have a better influence on slump flow. Sieve analysis and comparison revealed that the desulfurized slag had a more even particle size distribution. As a result, Specimens 1D, 2D, 3D, and 4D, which contained CFBC hydrated ash and desulfurized slag, presented better slump flow than the other specimens. In contrast, Specimens 1B, 2B, 3B, and 4B had the poorest slump flow values due to uneven aggregate grading. The increased flowability was probably due to the replacement of the aggregates, rough surface and fineness of the desulfurized slag particles [25].

### 3.2. Water-Soluble Chloride Ion Content

Table 4 displays the results of this test. Chloride ion content presented no significant increases as the proportion of CFBC hydrated ash in the aggregate increased from 15% to 45%. This indicates that the amount of CFBC hydrated ash does not influence the chloride ion content. The results showed that Specimens 1D, 2D, 3D, and 4D had the highest chloride ion content. All of these specimens contained desulfurized slag, which in itself had a higher chloride ion content of 0.17%. In contrast, the chloride ion contents of CFBC hydrated ash, coal bottom ash, and air-cooled blast furnace slag were 0.0143%, 0.0017%, and 0.0012%, respectively. Although the amount of desulfurized slag in the aggregates decreased as the proportion of CFBC hydrated ash increased, it did not significantly change the chloride ion content. We can therefore conclude that using desulfurized slag results in higher chloride ion content.

### 3.3. Ball Drop Test

Table 5 presents the ball drop test results at 24 h. As can be seen, at 24 h, the indentation diameters fell between 70 mm and 76 mm. When the proportion of CFBC hydrated ash increased from 15% to 45%, the indentation diameters did not change significantly, which means that changing the aggregate does not have any apparent influence on the ball drop test results. In conclusion, the results of this test all met CLSM requirements, so the CLSM mixtures can be used as backfill for roads.

### 3.4. Compressive Strength

**Figure 4:**
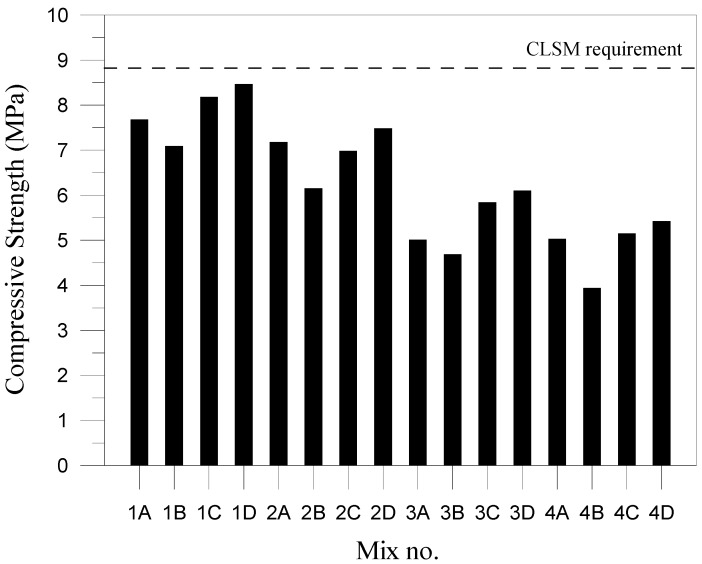
Compressive strength histograms.

This results in Figure 4 show that the specimens with the highest and lowest compressive strength are Specimens 1D, 2D, 3D, and 4D and Specimens 1B, 2B, 3B, and 4B, respectively. The results in these specimens show that the compressive strength declines as the proportion of CFBC hydrated ash increases from 15% to 45%. According to previous study [21], in which various amounts of CFBC ash were used to replace fine aggregates, a replacement amount of 25% presented a 25% drop in overall strength. This means that the compressive strength is inversely proportional to the amount of CFBC hydrated ash used. In conclusion, the specimens all showed compressive strength lower than stipulated by the Construction Commission of the Executive Yuan (8.82 MPa), thereby indicating that the mixtures can be applied to CLSM. However, the CFBC hydrated ash used in this study contains a high calcium ion content, which could be released upon alkali-activation and participate in the hydration reaction to form calcium silicate hydrate or calcium alumina silicate hydrate [10]. An increase in the replacement of by-product slag may result in a delay and decrease in the development of strength, which is consistent with the previous study [26]. 

### 3.5. Length Variation

Table 6 displays the changes in length displayed by the various specimens, all of which exhibited some swelling. At seven days, the difference was less than 0.1%. However, between day 7 and day 28, the swelling did not decelerate. On the contrary, some specimens presented a sharp increase in length. The specimens swelled regardless of the amount of CFBC hydrated ash or what aggregate the CFBC hydrated ash was paired with. The main cause of the swelling was the water-quenched blast furnace slag and the CFBC hydrated ash. The former contains free-CaO and free-MgO, which produce Ca(OH)_2_ and Mg(OH)_2_ in the presence of water and thereby increase the volume. As for the CFBC hydrated ash, it was stated that adding CFBC hydrated ash suppresses volumetric shrinkage [26].

## 4. Conclusions

This study investigated the application of CFBC hydration ash, combined bottom ash and CFB slag as recycled aggregates in CLSM. The results indicated that the specimens 1D, 2D, 3D, and 4D displayed the highest slump flow because they all contained desulfurized slag, which had a more even particle size distribution than the other types of aggregates. Desulfurized slag has higher chloride ion content; however, the amount of desulfurized slag used must depend on the regulations of where it is being applied to so as to prevent chloride ions from causing rebar corrosion.

CFBC hydrated ash resulted in the greatest compressive strength when paired with desulfurized slag but produced the lowest compressive strength when paired with coal bottom ash. Compressive strength decreased as the proportion of CFBC hydrated ash increased. The specimens all showed compressive strength lower than stipulated by the Construction Commission of the Executive Yuan (8.82 MPa), thereby indicating that the mixtures can be applied to CLSM. In addition, the indentation diameters derived in ball drop tests were all less than 76 mm, thereby meeting CLSM regulations and making the mixtures suitable as backfill for roads.

The primary binder in the mixtures in this study was water-quenched blast furnace slag, and the primary aggregate was CFBC hydrated ash. Both induce volumetric swelling, so the specimens in this study all displayed increased length, which must also be taken into consideration in future engineering applications as a backfill for roads or pipelines. This also reduced the cost of natural aggregates and the need to extract materials from the environment. This CLSM consisted of industrial by-products and recycled materials should be accepted and encouraged as the application of green materials.

## Figures and Tables

**Table 1:** Composition of CFBC hydrated ash.

Component	Content, wt %
Silicon dioxide (SiO_2_)	2.74
Aluminum oxide (Al_2_O_3_)	0.72
Ferric oxide (Fe_2_O_3_)	0.41
Potassium hydroxide (K_2_O)	0.34
Sodium oxide (Na_2_O)	0.13
Magnesium oxide (MgO)	1.38
Calcium sulfate dihydrate (CaSO_4_·2H_2_O)	33.83
Calcium sulfate (CaSO_4_)	12.02
Calcium carbonate (CaCO_3_)	24.49
Calcium hydroxide (Ca(OH)_2_)	16.91
Calcium oxide (CaO)	4.00
Carbon (C)	2.63

**Table 2:** Tested mixtures (kg/m^3^).

Specimen No.	Water-Quenched Blast Furnace Slag	Cement	CFBC Fly Ash	CFBC Hydrated Ash	Coal Bottom Ash	Air-Cooled Blast Furnace Slag	Desulfurized Slag	Water
1A	210	60	30	181	352	479	496	207
1B				181	1055	-	-	
1C				181	-	1436	-	
1D				181	-	-	1489	
2A				302	310	422	438	
2B				302	931	-	-	
2C				302	-	1267	-	
2D				302	-	-	1314	
3A				422	269	366	380	
3B				422	807	-	-	
3C				422	-	1098	-	
3D				422	-	-	1139	
4A				543	228	310	321	
4B				543	683	-	-	
4C				543	-	929	-	
4D				543	-	-	963	

**Table 3:** Slump flow of specimens.

Specimen No.	Slump Flow, cm
1A	42.5
1B	42.1
1C	44.4
1D	48.2
2A	44.0
2B	41.9
2C	48.3
2D	51.0
3A	48.2
3B	43.7
3C	50.0
3D	54.1
4A	48.7
4B	46.0
4C	51.5
4D	54.9

**Table 4:** Chloride ion content of specimens.

Specimen No.	Chloride Ion Content, kg/m^3^
1A	0.157
1B	0.023
1C	0.059
1D	0.314
2A	0.154
2B	0.027
2C	0.034
2D	0.318
3A	0.168
3B	0.028
3C	0.056
3D	0.272
4A	0.203
4B	0.033
4C	0.037
4D	0.263

**Table 5:** Indentation diameters on specimens in ball drop test.

Specimen No.	Indentation Diameter, mm
1A	73
1B	70
1C	72
1D	76
2A	75
2B	71
2C	71
2D	75
3A	75
3B	74
3C	76
3D	76
4A	76
4B	76
4C	76
4D	73

**Table 6:** Changes in length at various ages.

Specimen No.	Age, Days		
	7	14	28
1A	0.03%	0.08%	0.13%
1B	0.06%	0.08%	0.09%
1C	0.02%	0.06%	0.09%
1D	0.06%	0.10%	0.12%
2A	0.05%	0.18%	0.24%
2B	0.05%	0.08%	0.11%
2C	0.04%	0.16%	0.19%
2D	0.06%	0.08%	0.11%
3A	0.04%	0.10%	0.12%
3B	0.07%	0.10%	0.13%
3C	0.06%	0.07%	0.12%
3D	0.08%	0.12%	0.16%
4A	0.04%	0.06%	0.09%
4B	0.06%	0.10%	0.12%
4C	0.08%	0.09%	0.12%
4D	0.08%	0.10%	0.12%
